# Polytetrafluorethylene (PTFE) vs. Polyester (Dacron^®^) Grafts in Critical Limb Ischemia Salvage

**DOI:** 10.3390/ijerph20021235

**Published:** 2023-01-10

**Authors:** Eliza Russu, Adrian Vasile Mureșan, Adrian Dumitru Ivănescu, Réka Kaller, Daniela Elena Nedelea, Raluca Niculescu, Bogdan Andrei Cordoș, Ovidiu Aurelian Budișcă, Eliza Mihaela Arbănași, Emil Marian Arbănași

**Affiliations:** 1 Clinic of Vascular Surgery, Mureș County Emergency Hospital, 540136 Targu Mures, Romania; 2 Department of Vascular Surgery, George Emil Palade University of Medicine, Pharmacy, Science, and Technology of Targu Mures, 540139 Targu Mures, Romania; 3 Department of Anatomy, George Emil Palade University of Medicine, Pharmacy, Science, and Technology of Targu Mures, 540139 Targu Mures, Romania; 4 Doctoral School of Medicine and Pharmacy, George Emil Palade University of Medicine, Pharmacy, Sciences and Technology of Targu Mures, 540142 Targu Mures, Romania; 5 Department of Pathophysiology, George Emil Palade University of Medicine, Pharmacy, Science, and Technology of Targu Mures, 540139 Targu Mures, Romania; 6 Center for Advanced Medical and Pharmaceutical Research, George Emil Palade University of Medicine, Pharmacy, Sciences and Technology of Targu Mures, 540139 Targu Mures, Romania; 7 Veterinary Experimental Base, George Emil Palade University of Medicine, Pharmacy, Science, and Technology of Targu Mures, 540139 Targu Mures, Romania; 8 Department of Surgery, George Emil Palade University of Medicine, Pharmacy, Science, and Technology of Targu Mures, 540139 Targu Mures, Romania; 9 Faculty of Pharmacy, George Emil Palade University of Medicine, Pharmacy, Science, and Technology of Targu Mures, 540139 Targu Mures, Romania

**Keywords:** polytetrafluoroethylene, polyester, vascular surgery, bypass, critical limb ischemia

## Abstract

Background: Critical ischemia of the lower limbs refers to the last stages of peripheral arterial disease. It is characterized by resting discomfort or trophic disorders such as ulceration, skin necrosis, or gangrene in the lower limbs. Critical ischemia corresponds to Leriche–Fontaine (LF) stages III-IV and Rutherford stages 4–6. The purpose of this study was to observe the patency and postoperative complications of patients who have had infra-inguinal surgical revascularization and compare the results based on the kind of graft utilized. Methods: The present study was designed as an observational retrospective cohort study, including all patients from 2018 to 2019 diagnosed with severe ischemia of the lower limbs who were hospitalized at the Vascular Surgery Clinic of the County Emergency Clinical Hospital of Targu Mures. Results: Patients with a polytetrafluoroethylene (PTFE) graft had a higher incidence of chronic obstructive pulmonary disease (*p* = 0.01), stage III LF (70.41% vs. 55.29%), *p* = 0.03), and a lower incidence of stage IV LF (29.95% vs. 44.71%, *p* = 0.03). As for complications, the PTFE group showed a lower incidence of bypass thrombosis (29.59% vs. 44.71%; *p* = 0.03) and graft infection (9.18% vs. 21.18%; *p* = 0.02), but no statistical significance in the event of bleeding (*p* = 0.40). Regarding the outcomes, no statistical significance was seen for below-the-knee amputations or death. However, the PTFE group had a lower incidence of above-the-knee amputations (11.22% vs. 24.71%; *p* = 0.01). At multivariate analysis, the PTFE graft is an independent predictor of primary patency at 6, 12, and 24 months (OR: 2.15, *p* = 0.02; OR: 1.84, *p* = 0.04; and OR: 1.89, *p* = 0.03), as well as a protective factor against bypass thrombosis (OR: 0.52; *p* = 0.03), graft infection (OR: 0.37; *p* = 0.02), and above-the-knee amputation (OR: 0.38; *p* = 0.01).; Conclusions: According to this study’s findings, there were minor differences regarding the long-term patency, bypass thrombosis, graft infections, and above-the-knee amputations. In addition, the PTFE graft group had a higher incidence of primary patency at 6, 12, and 24 months, as well as a lower incidence of bypass thrombosis, graft infection, and above-the-knee amputations.

## 1. Introduction

Critical ischemia of the lower limbs refers to the last stages of the chronic progression of peripheral arterial disease. It is characterized by resting discomfort or trophic disorders such as ulceration, skin necrosis, or gangrene in the lower limbs, and corresponds to Leriche–Fontaine stages III-IV and Rutherford stages 4–6 [1]. If not treated immediately, it is associated with a high death rate and an even higher amputation rate at 6 months following diagnosis [2,3,4,5], and it is caused by predisposing factors and a variety of cardiovascular risk factors such as habitual smoking, diabetes, hypertension, hyperlipidemia, and obesity [6,7].

For patients with critical ischemia, surgical or endovascular revascularization is the first-line therapy. The selection of revascularization therapy is a matter that has lately received a lot of attention, and it takes numerous aspects into accounts, such as the type of damage and arterial location, the experience of the center, the patient’s medical state, or the existence of comorbidities. Moreover, multiple meta-analyses [8,9,10] comparing the two revascularization procedures have been reported in the literature.

Depending on the location of the artery lesion, the technical variations of surgical revascularization at the infra-inguinal level include the supra-genicular and infra-genicular femoropopliteal bypass, the popliteal–popliteal bypass, or the extra-anatomic femorofemoral bypass [11].

According to European Heart Association (EHA) and American Heart Association (AHA) recommendations, the first intention in patients with trophic damages and gangrenes is surgical revascularization, employing an autologous internal saphenous vein [12,13]. If this procedure is not viable because the vein is not suitable for extracting due to the risk of varicose development, a graft is a next option.

Grafts are used in vascular access surgery and surgical revascularization [14,15,16,17,18,19,20,21]. Numerous studies have been conducted in recent years to assess graft biocompatibility and infection resistance [22,23,24]. Polytetrafluoroethylene (PTFE), known as Gore-Tex, and polyethylene terephthalate (Dacron), known as the textile graft, are the most commonly used allografts in vascular surgery.

Since all prosthetic graft components have a distinct impact on graft function, porosity, compliance, and flow surface, each type is unique and has its own set of characteristics [22]. Crystalline and hydrophobic polymeric molecules are employed in the two most common synthetic grafts. Dacron grafts are clinically accessible in woven or knitted forms. The multifilament threads of the woven graft are arranged in an over-and-under manner, leading to reduced porosity. As a result, these grafts have less through-bleeding, which is beneficial in some circumstances. The threads in the knitted variety are looped to interlock in a chain-like pattern, resulting in higher porosity and radial distensibility, as well as improved tissue integration.

Preclotting is essential due to the high porosity of the grafts, and materials such as gelatin, collagen, or albumin are used to fill the spaces. Some manufacturers employ low formaldehyde concentrations to cross-link the gelatin or collagen used to seal the pores, allowing them to organically dissolve in about two weeks. Other producers utilize glutaraldehyde to cross-link albumin, which permits the albumin to break down in approximately two months. Crimping increases the elasticity and kink resistance of Dacron grafts. However, it has been reported that this technique causes an uneven interna and luminal surface, as well as an increased thrombosis. A textile graft, especially one that is knitted, tends to dilate, as was the case with the first generation of grafts made using the double-velour technique, which used trilobal filaments. The textile graft is primarily used in aortoiliac revascularization and abdominal aortic aneurysms [25,26,27].

After the Dacron graft is inserted, a fibrin layer forms on the blood-contacting surface. This fibrin layer extends from the anastomosis sites to the graft section’s center. No matter how inert the components in the grafts’ composition are, these will still be perceived as foreign. After the protein adsorption, platelet deposition, and the infiltration of neutrophils and monocytes, the smooth muscle cell proliferation is completed, or so-called “neointimal” or “pseudo-intimal” hyperplasia, which will eventually produce the graft’s internal “coating”, resembling the arterial intimal layer.

The PTFE molecule is biologically stable, and due to the electronegative surface, the interaction of blood cells with the prosthesis is minimal. Regardless of how many years have passed since implantation, the absence of the pseudo-intimal layer in the mid-graft portion is a known characteristic of this type of graft. The carbon covering the PTFE graft improves electronegativity and combats thrombosis, and the design of this graft can be adjusted to mimic a vein “cuff” with the inner structure in the pre-cuffed region for vascular access and infrapopliteal revascularization. In addition, rings or coils can be added to the exterior of the PTFE graft to prevent it from collapsing, making it suitable for use in an extra-anatomical position. Moreover, for improved performance, both kinds of grafts can be heparin-bonded. Antibiotic or silver-bonded textile grafts can also be used to facilitate bacteria-free recovery. However, the structural features of PTFE make it more resistant to germs.

The purpose of this study is to observe the patency and postoperative complications of patients who have had infra-inguinal surgical revascularization and compare these results based on the kind of graft utilized.

## 2. Materials and Methods

### 2.1. Study Design

An observational retrospective cohort study was carried out, including all patients from 2018 to 2019 diagnosed with severe ischemia of the lower limbs who were hospitalized at the Vascular Surgery Clinic of the County Emergency Clinical Hospital of Targu Mures, Romania. Patients’ data were collected using observation sheets and the computerized medical system, and they were followed up with postoperative visits to the specialized outpatient clinic.

### 2.2. Data Collection

This study included 183 patients who had significant lower limb ischemia, infra-inguinal arterial lesions, and required surgical revascularization. Patients with stage III-IV Leriche–Fontaine peripheral artery disease with an indication for surgical revascularization and the inability to use the autologous internal saphenous vein for revascularization were among the selection criteria. Individuals having a history of surgical or endovascular revascularization of the afflicted limb before admission, as well as those with hematological illnesses, recent tumoral status, or systemic inflammatory disease, were not eligible for the research.

The demographic data and the following comorbidities were extracted from the patient’s medical history in the hospital’s electronic database: arterial hypertension (AH), ischemic heart disease (IHD), atrial fibrillation (AF), myocardial infarction (MI), chronic heart failure (CHF), chronic obstructive pulmonary disease (COPD), chronic kidney disease (CKD), cerebrovascular accident (CVA), type 2 diabetes (T2D), chronic venous insufficiency (CVI), peripheral artery disease (PAD), dyslipidemia, tobacco usage, and obesity.

The following laboratory results were collected on the first day of hospitalization: complete blood counts (lymphocyte, monocyte, neutrophil, and platelets), hemoglobin, hematocrit, blood urea nitrogen (BUN), creatinine, e-glomerular filtration rate (eGFR), alanine aminotransferase (ALT), aspartate aminotransferase (AST), total bilirubin, sodium, potassium, international normalized ratio (INR), and activated partial thromboplastin time (aPTT). Regarding the systemic inflammation, we used the complete blood counts and calculated the hematological ratios: monocyte-to-lymphocyte ratio (MLR = monocytes/lymphocytes), neutrophil-to-lymphocyte ratio (NLR = neutrophils/lymphocytes), platelet-to-lymphocyte ratio (PLR = platelets/lymphocytes), and systemic inflammatory index (SII = neutrophils x platelets/lymphocytes). Numerous articles published recently in the literature on cardiovascular disease and other chronic diseases revealed the prognostic role of these markers in poor outcomes [28,29,30,31,32,33,34,35,36,37]. Furthermore, for trophic lesions, we used the Society for Vascular Surgery’s classification system, which was based on the characteristics and presence of the wound, severity of ischemia, and severity of foot infection (WIfI classification), with each component of classification receiving 0 to 3 points based on the WIfI score [38].

### 2.3. Revascularization Technique

Participants in the study underwent revascularization surgery, with the following procedures performed depending on the location of the arterial lesion: above-the-knee femoropopliteal bypass (AK FP bypass), below-the-knee femoropopliteal bypass (BK FP bypass), or extra-anatomical femorofemoral bypass (FF bypass). All patients had the same postoperative treatment, which included anti-aggregates, anticoagulants, and statins. Moreover, all patients were operated on by the same surgeon to minimize the bias.

The primary goal of this research was to track the patency and postoperative complications of patients receiving infra-inguinal surgical revascularization and evaluate the results based on the graft type employed. We also evaluated relevant complications and bypass patency at 1, 6, 12, and 24 months after surgery. The follow-up patency was measured using ultrasonography, and in the case of uncertainty or impossibility, Computed Tomography Angiography was performed.

### 2.4. Statistical Analysis

Data are presented as mean ± SD if customarily distributed and median (interquartile range) if non-parametrically distributed. Differences between groups were tested using a two-tailed Student’s t-test or Mann–Whitney U test appropriate for two-group comparisons. Categorical variables were compared with the χ2-test. In terms of long-term patency, we compared the two types of grafts using the Kaplan–Meier curve and the long-rank test. All *p*-values are two-tailed, with a *p* < 0.05 considered statistically significant. Statistical analysis was performed using SPSS for Windows version 28.0 (SPSS, Inc., Chicago, IL, USA).

## 3. Results

The patients were divided into two groups based on the type of prosthesis used: in the first group, we included the patients with Dacron prosthesis (85 cases), and in the second group, we included the patients with the PTFE type (98 cases). Analyzing the patients included in the study, we had an average age of 69.2 years, with patients aged between 51 and 92 years and 75.41% of the patients being males. Regarding the Leriche–Fontaine classification, 63.38% of the patients were in stage III, and 36.61% were in stage IV. Among the comorbidities of the patients, the highest incidence was AH at 85.25% (156 patients), followed by IHD at 82.51% (151 patients), CHF in 63.93% of the cases, T2D at 48.63%, MI at 33.88%, AF at 25.68%, and COPD in 22.95% of cases. In 24.5% of the cases, patients had a history of CVA, 21.86% of patients had CKD, and 27.87% had CVI. As cardiovascular risk factors, 77.05% of patients had a history of long-term smoking, 65.03% had hyperlipidemia, and 40.44% were overweight (Table 1).

The incidence of comorbidities and risk factors was compared across the two study groups, but no statistically significant variations were found in mean age (69.79 vs. 68.52 years old, *p* = 0.31), AH (83.67% vs. 87.06%, *p* = 0.52), IHD (83.67% vs. 81.18%, *p* = 0.65), AF (24.49% vs. 27.06%, *p* = 0.69), MI (33.67% vs. 34.12%, *p* = 0.94), CHF (61.22% vs. 67.06%, *p* = 0.41), CKD (23.47% vs. 20%, *p* = 0.57), T2D (51.02% vs. 45.88%, *p* = 0.48), CVA (23.47% vs. 25.88%, *p* = 0.70), CVI (22.45% vs. 34.12%, *p* = 0.08), tobacco use (77.05% vs. 75.29%, *p* = 0.59), hyperlipidaemia (61.22% vs. 69.41%, *p* = 0.24), and obesity (40.82% vs. 40 %, *p* = 0.91).

Statistically significant differences were found between the two groups correlated to critical ischemia, in stage III LF (70.41% vs. 55.29%, *p* = 0.03), and stage IV LF (29.95% vs. 44.71%, *p* = 0.03). A statistical significance was also found in COPD patients (29.59% vs. 15.29%, *p* = 0.01). In terms of WIfI classification, there were lower incidences of wound grade 0 (*p* = 0.03), ischemia grade 3 (*p* = 0.02), and foot infection grade 0 (*p* = 0.03) in the Dacron group, as shown in Table 1.

Only the INR was found to be higher in the Dacron group (*p* = 0.03) in terms of laboratory results. Table 2 shows that there were no statistically significant differences between the two groups for the remaining laboratory dates analyzed.

As for surgical revascularization, depending on the location of the arterial injury, in 62.29% (114 patients) of the cases, an AK FP bypass was performed, followed by a BK FP bypass in 22.95% (42 patients), and an FF bypass in 14.75% (27 patients). At 1-month post-operation, we registered a patency of 90.16%, which decreased to 73.77% at 6 months, 57.38% at 12 months, and 50.82% at 24 months following surgery for all patients. Graft thrombosis was the most frequent complication in the studied patients, accounting for 36.61% of the cases, followed by graft infection in 14.75%, and bleeding in 7.65% of cases. Amputations of the affected limb were performed for all poor outcomes or advanced trophic lesions. These were performed above-the-knee in 17.49% of the patients who benefited from surgical revascularization and below-the-knee in 8.74%. During the 24-month follow-up period, 21 deaths were recorded.

After one month following surgery, no statistically significant differences in patency were observed for any type of graft used (90.82% vs. 89.41%; *p* = 0.75), but significant differences were observed at 6 months (80.61% vs. 65.88%; *p* = 0.02), 12 months (64.29% vs. 49.41%; *p* = 0.04), and 24 months (58.16% vs. 42.35%; *p* = 0.03), respectively.

In terms of complications, the PTFE group had a lower incidence of bypass thrombosis (29.59% vs. 44.71%; *p* = 0.03) and graft infection (9.18% vs. 21.18%; *p* = 0.02), but no statical significance was found in cases of bleeding (*p* = 0.40). Regarding the outcomes, no statistical significance was registered in the case of below-the-knee amputations or death. However, the PTFE group had a lower incidence of above-the-knee amputations (11.22% vs. 24.71%; *p* = 0.01) (Table 3).

To increase the accuracy of the data, the patency and complications were evaluated separately depending on the type of intervention done. Furthermore, because only a limited number of patients required an extra-anatomic FF bypass, only those who benefited from AK and BK FP bypasses were studied (Table 4, Table 5).

For the patients who benefited from an AK FP bypass, no statistical differences were seen between the Leriche–Fontaine stage III (74.03% vs. 64.86%; *p* = 0.31) and stage IV (25.97% vs. 35.14%; *p* = 0.31). Moreover, no statistically significant differences were found between the 1-month bypass patency (90.91% vs. 97.29%; *p* = 0.23), 6 months (79.22% vs. 72.97%; *p* = 0.45), 12 months (63.63% vs. 56.76%; *p* = 0.47), and 24 months (57.14% vs. 54.05%; *p* = 0.75). Graft infection occurred at a rate of 10.39% in the first group and 21.62% in the second group (*p* = 0.11). Furthermore, some patients presented with postoperative bleeding (6.49% vs. 5.41%; *p* = 0.82), and in terms of outcomes, there were no differences between amputations performed below-the-knee (3.9% vs. 5.41%; *p* = 0.71) and deaths (3.9% vs. 13.51%; *p* = 0.07). However, the PTFE group had a lower risk of above-the-knee amputation (11.69% vs. 27.03%; *p* = 0.04) (Table 4).

For the BK FP bypass group of patients, there were no statistically significant differences in the 1-month patency (89.47% vs. 82.62%; *p* = 0.53), 6 months (84.21% vs. 65.22%; *p* = 0.17), or 12-month patency (63.16% vs. 39.13%; *p* = 0.12). However, at 24 months, we had higher patency in the Dacron group (57.89% vs. 26.09%; *p* = 0.04). There were no significant differences in postoperative complications and outcomes between patients who received an BK FP bypass with Dacron or PTFE prosthesis (Table 5).

A multivariate analysis was used to determine the association between the graft type and all complications and outcomes at 24 months following the revascularization. The PTFE graft is an independent predictor of primary patency at 6, 12, and 24 months (OR:2.15, *p* = 0.02; OR:1.84, *p* = 0.04; and OR: 1.89, *p* = 0.03), as well as a protective factor against bypass thrombosis (OR: 0.52; *p* = 0.03), graft infection (OR:0.37; *p* = 0.02), and above-the-knee amputation (OR:0.38; *p* = 0.01), as seen in Table 6.

**Figure 1:**
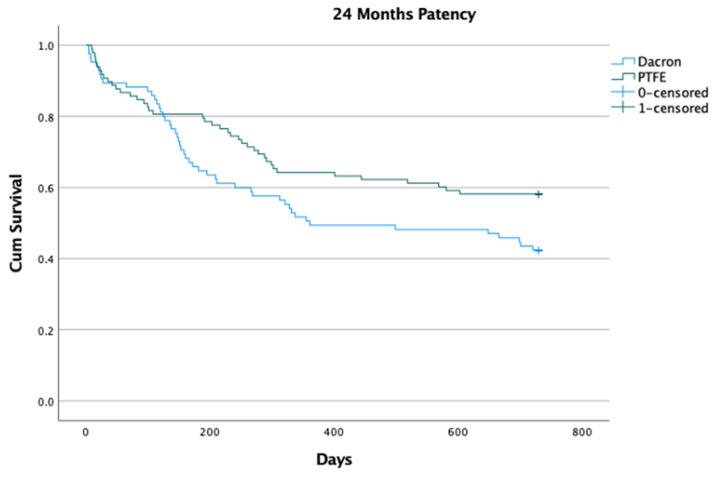
Kaplan–Meier 24-month patency curves for PTFE and Dacron groups in all patients (*p* = 0.043).

The Kaplan–Meier chart for the 24-month patency based on the type of graft for all patients is shown in Figure 1.

## 4. Discussion

The main findings of this study are slight variations in long-term patency, bypass thrombosis, graft infections, and above-the-knee amputations, with the PTFE graft group having a higher incidence of primary patency at 6, 12, and 24 months, as well as lower incidences of bypass thrombosis, graft infections, and above-the-knee amputations. Moreover, there were no statical differences for 1-month patency, bleeding, below-the-knee amputations, and mortality. Furthermore, regarding the type of bypass performed, in above-the-knee FP bypasses, there was a higher incidence of above-the-knee amputations (*p* = 0.04) with no other statistical difference, and for below-the-knee FP bypass, there was higher 24-month patency in the PTFE group (*p* = 0.04).

Many studies over the last decades have been conducted to investigate the structural properties of Dacron and PTFE. Greisler [27] and King et al. [39] have studied the material imperfections and the causes of graft failures [40,41,42,43]. The collagen coating, the albumin and carbon impregnation, and the Rifampicin impregnation have been also widely studied [44,45,46].

Multicenter studies have tried to compare the performance of the two grafts when functioning in each position. In the study by Prager [47], from the patency point of view, there were no statistically significant differences between patients with Dacron and those with PTFE prostheses. Other studies published in the literature, including patients with above-the-knee and below-the-knee bypasses, did not find statistically significant differences between the two grafts [48,49,50,51]. In the paper published by Robinson et al., the Dacron prosthesis group had a patency of 70% at 12 months and 56% at 24 months, whereas the PTFE group had a patency of 72% at 12 months and 52% at 24 months [49]. Moreover, Devine and McCollum published a second investigation, which reported that the group having the Dacron prosthesis had a patency of 71% at 12 months and 54% at 36 months, whereas the group receiving the PTFE prosthesis had a patency of 62% at 12 months and 44% at 24 months [50]. Following three years of monitoring for each type of graft in the Post et al. article, the percentage of bypasses was 64% for patients with Dacron and 61% for those with PTFE [51], which is similar to our study’s findings.

In the paper published by Green et al., they compared the patency of the two grafts implanted in an AK position, showing no statistically significant differences between the two, reporting a patency at 12 months of 65% in the Dacron prostheses group and 63% in the PTFE group [52]. Robinson and Fletcher analyzed the patency of AK and BK FP bypasses and obtained higher patency in patients with PTFE prostheses, presenting 71% at 6 months and 56% at 12 months, compared to 50% at 6 months and 36% at 12 months in patients with Dacron [53]. Contrarily, in the study published by Jensen et al., a higher patency rate was obtained in patients with Dacron-type prostheses, with 70% at 24 months compared to 57% for patients with PTFE [54].

Choosing the type of graft for surgical revascularization is currently a significant issue for surgeons. Despite the lack of a current meta-analysis, the results of our study and the literature highlights show no significant differences between the two types of grafts used for infra-inguinal surgical revascularization. Therefore, even though PTFE prostheses are mainly used in extra-anatomical femoral-femoral bypasses, the most suitable prosthesis type remains the surgeon’s choice. Another critical aspect of the procedure is the cost, with Dacron-type prostheses being less expensive in many countries.

However, this study has some significant limitations, reporting a small number of patients from a single center and most patients being male, so the results cannot be generalized. Moreover, the 24-month follow-up is a short interval to state broad conclusions. In addition, the study’s retrospective design is another limitation. Prospective, multicentric, long-term follow-up studies are recommended in the future.

## 5. Conclusions

Regardless of the type of bypass performed, there were minor statistically significant variations between the two types of a prosthesis in terms of patency, frequency of difficulties, and the rate of amputation. As a result, when it comes to bypass choice, the surgeon has the final decision. A multicenter research study should be conducted for each kind of bypass in the future, and patency should be monitored for a longer period to improve statistical accuracy.

## Figures and Tables

**Table 1:** Characteristics, demographics, comorbidities, and risk factors of patients.

Characteristics	All Patients (n = 183)	PTFE Group (n = 98)	Dacron Group (n = 85)	*p* Value
Age (years) mean ± SD	69.2 ± 8.54	69.79 ± 8.38	68.52 ± 8.71	0.31
Sex (M) (%, no)	75.41% (138)	79.59% (78)	70.53% (60)	0.16 (0.61; 0.31–1.21)
Leriche–Fontaine Classification				
Stage III LF (%, no)	63.38% (116)	70.41% (69)	55.29% (47)	0.03 (0.51; 0.28–0.95)
Stage IV LF (%, no)	36.61% (67)	29.59% (29)	44.71% (38)	0.03 (1.92; 1.04–3.53)
Wifi Classification				
Wound grade				
0	63.38% (116)	70.41% (69)	55.29% (47)	0.03
1	21.86% (40)	18.37% (18)	25.88% (22)	0.22
2	10.93% (20)	9.18% (9)	12.94% (11)	0.41
3	2.04% (7)	2.04% (2)	5.88% (5)	0.19
Ischemia severity				
0	18.58% (34)	14.29% (14)	23.53% (20)	0.11
1	31.15% (57)	31.63% (31)	30.59% (26)	0.87
2	37.16% (68)	35.71% (35)	35.71% (33)	0.66
3	13.11% (24)	18.37% (18)	7.06% (6)	0.02
Foot infection grade				
0	63.38% (116)	70.41% (69)	55.29% (47)	0.03
1	82.51% (151)	83.67% (82)	81.18% (69)	0.07
2	25.68% (47)	24.49% (24)	27.06% (23)	0.24
3	85.25% (156)	83.67% (82)	87.06% (74)	0.55
Comorbidities				
AH (%, no)	85.25% (156)	83.67% (82)	87.06% (74)	0.52 (1.31; 0.57–3.008)
IHD (%, no)	82.51% (151)	83.67% (82)	81.18% (69)	0.65 (0.84; 0.39–1.80)
AF (%, no)	25.68% (47)	24.49% (24)	27.06% (23)	0.69 (1.14; 0.58–2.22)
MI (%, no)	33.88% (62)	33.67% (33)	34.12% (29)	0.94 (1.02; 0.55–1.88)
CHF (%, no)	63.93% (117)	61.22% (60)	67.06% (57)	0.41 (1.28; 0.70–2.36)
COPD (%, no)	22.95% (42)	29.59% (29)	15.29% (13)	0.02 (0.42; 0.20–0.89)
CKD (%, no)	21.86% (40)	23.47% (23)	20% (17)	0.57 (0.81; 0.40–1.65)
CVA (%, no)	24.59% (45)	23.47% (23)	25.88% (22)	0.70 (1.13; 0.58–2.23)
T2D (%, no)	48.63% (89)	51.02% (50)	45.88% (39)	0.48 (0.81; 0.45–1.45)
CVI (%, no)	27.87% (51)	22.45% (22)	34.12% (29)	0.08 (1.78; 0.93–3.43)
Risk Factors				
Obesity (%, no)	40.44% (74)	40.82% (40)	40% (34)	0.91 (0.96; 0.53–1.74)
Hyperlipidemia (%, no)	65.03% (119)	61.22% (60)	69.41% (59)	0.24 (1.43; 0.77–2.65)
Tobacco (%, no)	77.05% (141)	78.57% (77)	75.29% (64)	0.59 (0.83; 0.41–1.65)

AH = arterial hypertension; IHD = ischemic heart disease; AF = atrial fibrillation; MI = myocardial infarction; CHF = chronic heart failure; COPD = chronic obstructive pulmonary disease; CKD = chronic kidney disease; T2D = type 2 diabetes; CVA = cerebrovascular accident.

**Table 2:** Laboratory data for all patients, PTFE group and Dacron group.

Variables (Mean ± SD)	All Patients (n = 183)	PTFE Group (n = 98)	Dacron Group (n = 85)	*p* Value
Hemoglobin g/dL	13.67 + 1.72	13.55 + 1.62	13.8 + 1.84	0.28
Hematocrit %	41.68 + 5.11	41.25 + 4.68	42.18 + 5.55	0.20
Glucose mg/dL	119.55 + 46.02	119.08 + 49.68	120.09 + 41.7	0.17
ALT u/L	29.99 + 3.47	31.54 + 4.29	28.19 + 2.25	0.27
AST u/L	35.91 + 4.33	38.28 + 4.97	33.17 + 3.45	0.34
Total bilirubin mg/dL	0.48 + 0.24	0.47 + 0.25	0.49 + 0.22	0.07
BUN mg/dL	42.39 + 20.16	40.73 + 17.31	44.3 + 22.97	0.30
Creatinine mg/dL	1.03 + 071	1.08 + 0.90	0.96 + 0.38	0.07
GFR (mL/min/1.73 m^2^)	77.28 + 23.56	76.85 + 22.6	77.78 + 24.74	0.46
K mmol\L	4.13 + 0.5	4.15 + 0.51	4.1 + 0.48	0.32
Na mmol\L	140.16 + 3.87	139.9 + 3.72	140.47 + 4.03	0.09
INR	1.11 + 0.17	1.08 + 0.16	1.14 + 0.19	0.03
APTT (sec)	30.66 + 5.82	30.7 + 5.87	30.62 + 5.84	0.48
Monocyte × 10^3^/uL	2.19 + 0.73	2.24 + 0.65	2.14 + 0.81	0.11
Lymphocytes × 10^3^/uL	0.58 + 0.15	0.58 + 0.12	0.59 + 0.18	0.46
Monocyte × 10^3^/uL	5.59 + 1.82	5.49 + 1.71	5.72 + 1.94	0.26
Neutrophils × 10^3^/uL	194.29 + 49.59	194.27 + 49.28	194.32 + 50.24	0.48
PLT × 10^3^/uL	0.30 + 0.13	0.28 + 0.10	0.32 + 0.15	0.10
MLR	2.90 + 1.53	2.66 + 1.13	3.17 + 1.86	0.07
NLR	100.11 + 46.31	93.59 + 34.29	107.61 + 56.42	0.18
PLR	13.67 + 1.72	13.55 + 1.62	13.8 + 1.84	0.28

**Table 3:** Type of surgery, complications, and outcomes of all patients enrolled in the study.

	All (n = 183)	PTFE Group (n = 98)	Dacron Group (n = 85)	*p* Value
Type of surgery				
AK FP bypass, (no, %)	114 (62.29%)	77 (78.57%)	37 (43.53%)	<0.0001 (0.21; 0.11–0.40)
BK FP bypass, (no, %)	42 (22.95%)	19 (19.39%)	23 (27.06%)	0.22 (1.54; 0.77–3.08)
FF bypass, (no, %)	27 (14.75%)	2 (2.04%)	25 (29.41%)	0.0001 (20.01; 4.57–87.5)
Patency of bypass				
1 month, (%, no)	90.16% (165)	90.82% (89)	89.41% (76)	0.75 (0.85; 0.32–2.26)
6 months, (%, no)	73.77% (135)	80.61% (79)	65.88% (56)	0.02 (0.46; 0.23–0.90)
12 months, (%, no)	57.38% (105)	64.29% (63)	49.41% (42)	0.04 (0.54; 0.29–0.98)
24 months, (%, no)	50.82% (93)	58.16% (57)	42.35% (36)	0.03 (0.52; 0.29–0.95)
Outcomes				
Bypass thrombosis (%, no)	36.61% (67)	29.59% (29)	44.71% (38)	0.03 (1.92; 1.04–3.53)
Graft infection (%, no)	14.75% (27)	9.18% (9)	21.18% (18)	0.02 (2.65; 1.12–6.28)
Bleeding (%, no)	7.65% (14)	9.18% (9)	5.85% (5)	0.40 (0.61; 0.19–1.92)
Above-the-knee amputation, (%, no)	17.49% (32)	11.22% (11)	24.71% (21)	0.01 (2.59; 1.16–5.76)
Below-the-knee amputation, (%, no)	8.74% (16)	7.14% (7)	10.59% (9)	0.41 (1.53; 0.54–4.32)
Death, (%, no)	11.47% (21)	8.16% (8)	14.11% (13)	0.13 (2.03; 0.79–5.16)

AK FP bypass = above-the-knee femoropopliteal bypass; BK FP bypass = below-the-knee femoropopliteal bypass; FF bypass = femoro-femoral bypass.

**Table 4:** Leriche–Fontaine classification, patency, complications, and outcomes of AK FP bypass patients.

	PTFE Group (n = 77)	Dacron Group (n = 37)	*p* Value
Stage III LF (%, no)	74.03% (57)	64.86% (24)	0.31 (0.64; 0.27–1.50)
Stage IV LF (%, no)	25.97% (20)	35.14% (13)	0.31 (1.54; 0.66–3.59)
Patency of bypass			
1 month (%, no)	90.91% (70)	97.29% (36)	0.23 (3.60; 0.42–30.41)
6 months (%, no)	79.22% (61)	72.97% (27)	0.45 (0.70; 0.28–1.76)
12 months (%, no)	63.64% (49)	56.76% (21)	0.48 (0.75; 0.33–1.66)
24 months (%, no)	57.14% (44)	54.05% (20)	0.75 (0.88; 0.40–1.94)
Outcomes			
Bypass thrombosis (%, no)	31.17% (24)	35.14% (13)	0.67 (1.19; 0.52–2.74)
Graft infection (%, no)	10.39% (8)	21.62% (8)	0.11 (2.37; 0.81–6.94)
Bleeding (%, no)	6.49% (5)	5.41% (2)	0.82 (0.82; 0.15–4.45)
Above-the-knee amputation (%, no)	11.69% (9)	27.03% (10)	0.04 (2.79; 1.02–7.64)
Below-the-knee amputation (%, no)	3.9% (3)	5.41% (2)	0.71 (1.40; 0.22–8.82)
Deaths (%, no)	3.9% (3)	13.51% (5)	0.07 (3.85; 0.86–17.10)

**Table 5:** Leriche–Fontaine classification, patency, complications, and outcomes of BK FP bypass patients.

	PTFE Group (n = 19)	Dacron Group (n = 23)	*p* Value
Stage III LF (%, no)	57.89% (11)	26.09% (6)	0.04 (0.25; 0.06–0.94)
Stage IV LF (%, no)	42.11% (8)	73.91% (17)	0.04 (3.89; 1.05–14.32)
Patency of bypass			
1 month (%, no)	89.47% (17)	82.62% (19)	0.53 (0.55; 0.09–3.44)
6 months (%, no)	84.21% (16)	65.22% (15)	0.17 (0.35; 0.07–1.57)
12 months (%, no)	63.16% (12)	39.13% (9)	0.12 (0.37; 0.10–1.31)
24 months (%, no)	57.89% (11)	26.09% (6)	0.04 (0.25; 0.06–0.94)
Outcomes			
Bypass thrombosis (%, no)	52.63% (9)	47.83% (11)	0.98 (1.01; 0.25–4.02)
Graft infection (%, no)	31.57% (6)	30.43% (7)	0.93 (0.94; 0.25–3.52)
Bleeding *(*%, no)	26.31% (5)	8.7% (2)	0.14 (0.26; 0.04–1.57)
Above-the-knee amputation (%, no)	31.57% (6)	21.74% (5)	0.60 (0.60; 0.15–2.40)
Below-the-knee amputation (%, no)	21.05% (4)	26.09% (6)	0.70 (1.32; 0.31–5.60)
Death (%, no)	15.78% (3)	17.39% (4)	0.88 (1.12; 0.21–5.70)

**Table 6:** Multivariate analysis regarding the type of graft and all outcomes.

Primary Patency	Dacron Group			PTFE Group		
	OR	95% CI	*p* Value	OR	95% CI	*p* Value
1 month	0.85	0.32–2.26	0.75	1.17	0.44–3.09	0.75
6 months	0.46	0.23–0.91	0.02	2.15	1.09–4.21	0.02
12 months	0.54	0.30–0.98	0.04	1.84	1.01–3.33	0.04
24 months	0.52	0.29–0.95	0.03	1.89	1.05–3.40	0.03
**Complications**	**OR**	**95% CI**	***p* Value**	**OR**	**95% CI**	***p* Value**
Bypass thrombosis	1.92	1.04–3.53	0.03	0.52	0.28–0.95	0.03
Graft infection	2.65	1.12–6.28	0.02	0.37	0.15–0.89	0.02
Bleeding	0.61	0.19–1.92	0.40	1.61	0.52–5.03	0.40
Above-the-knee amputation	2.59	1.16–5.76	0.01	0.38	0.17–0.85	0.01
Below-the-knee amputation	1.53	0.54–4.32	0.41	0.65	0.23–1.82	0.41
Death	0.37	0.03–3.69	0.40	2.65	0.27–25.89	0.40

## Data Availability

Not applicable.

## References

[1] Kinlay S. (2016). Management of Critical Limb Ischemia. Circ. Cardiovasc. Interv..

[2] Murabito J.M., Evans J.C., Nieto K., Larson M.G., Levy D., Wilson P.W.F. (2002). Prevalence and Clinical Correlates of Peripheral Arterial Disease in the Framingham Offspring Study. Am. Heart J..

[3] Stoyioglou A., Jaff M.R. (2004). Medical Treatment of Peripheral Arterial Disease: A Comprehensive Review. J. Vasc. Interv. Radiol..

[4] Abu Dabrh A.M., Steffen M.W., Undavalli C., Asi N., Wang Z., Elamin M.B., Conte M.S., Murad M.H. (2015). The Natural History of Untreated Severe or Critical Limb Ischemia. J. Vasc. Surg..

[5] Norgren L., Hiatt W.R., Dormandy J.A., Nehler M.R., Harris K.A., Fowkes F.G.R. (2007). Inter-Society Consensus for the Management of Peripheral Arterial Disease (TASC II). J. Vasc. Surg..

[6] Steg P.G., Bhatt D.L., Wilson P.W.F., D’Agostino R., Ohman E.M., Röther J., Liau C.-S., Hirsch A.T., Mas J.-L., Ikeda Y. (2007). One-Year Cardiovascular Event Rates in Outpatients With Atherothrombosis. JAMA.

[7] Caro J., Migliaccio-Walle K., Ishak K.J., Proskorovsky I. (2005). The Morbidity and Mortality Following a Diagnosis of Peripheral Arterial Disease: Long-Term Follow-up of a Large Database. BMC Cardiovasc. Disord..

[8] Bradbury A.W., Adam D.J., Bell J., Forbes J.F., Fowkes F.G.R., Gillespie I., Ruckley C.V., Raab G.M. (2010). Bypass versus Angioplasty in Severe Ischaemia of the Leg (BASIL) Trial: Analysis of Amputation Free and Overall Survival by Treatment Received. J. Vasc. Surg..

[9] Romiti M., Albers M., Brochado-Neto F.C., Durazzo A.E.S., Pereira C.A.B., De Luccia N. (2008). Meta-Analysis of Infrapopliteal Angioplasty for Chronic Critical Limb Ischemia. J. Vasc. Surg..

[10] Albers M., Romiti M., Brochado-Neto F.C., Pereira C.A.B. (2005). Meta-Analysis of Alternate Autologous Vein Bypass Grafts to Infrapopliteal Arteries. J. Vasc. Surg..

[11] Slovut D.P., Lipsitz E.C. (2012). Surgical Technique and Peripheral Artery Disease. Circulation.

[12] Anderson J.L., Halperin J.L., Albert N., Bozkurt B., Brindis R.G., Curtis L.H., DeMets D., Guyton R.A., Hochman J.S., Kovacs R.J. (2013). Management of Patients With Peripheral Artery Disease (Compilation of 2005 and 2011 ACCF/AHA Guideline Recommendations). J. Am. Coll. Cardiol..

[13] Conte M.S., Bradbury A.W., Kolh P., White J.V., Dick F., Fitridge R., Mills J.L., Ricco J.-B., Suresh K.R., Murad M.H. (2019). Global Vascular Guidelines on the Management of Chronic Limb-Threatening Ischemia. Eur. J. Vasc. Endovasc. Surg..

[14] Muresan V.A., Cosarca M.C., Russu E., Niculescu R., Zăgan C.A. (2021). A Rare Case of Abdominal Aortic Aneurysm with Ureteral Compression. J. Interdiscip. Med..

[15] Mocian A., Russu E., Kaller R., Mureșan A. (2019). Aorto-Mesenteric Bypass for the Treatment of Chronic Mesenteric Ischemia. J. Interdiscip. Med..

[16] Muresan V.A., Cosarca M.C., Russu E., Niculescu R., Soimu M. (2021). Ilio-Deep Femoral Bypass—An Alternative Treatment Strategy to Critical Limb Ischemia (CLI). J. Interdiscip. Med..

[17] Russu E. (2011). Rolul By-Pass-ului extra-anatomic in managementul ischemiei membrelor inferioare. Ph.D. Thesis.

[18] Eliza R., Toma L., Mureșan A., Preda R., Oroșan S., Constantinescu F., Grigorescu B. (2010). Risk Scoring Systems Used in the Multidisciplinary decision of Extra-Anatomic By-passes. Acta Med. Marisiensis.

[19] Kaller R., Mureșan A.V., Popa D.G., Arbănași E.-M., Russu E. (2021). Fatal Aortoduodenal Fistula Caused by a Ruptured Abdominal Aortic Aneurysm—A Case Report. J. Cardiovasc. Emergencies.

[20] Kaller R., Mureșan A.V., Arbănași E.M., Arbănași E.M., Kovács I., Horváth E., Suciu B.A., Hosu I., Russu E. (2022). Uncommon Surgical Management by AVF between the Great Saphenous Vein and Anterior Tibial Artery for Old Radiocephalic AVF Failure. Life.

[21] Arbanasi E.M., Russu E., Muresan A.V., Arbanasi E.M., Kaller R. (2021). Ulnar-Basilic Arteriovenous Fistula with Multilocular Gigantic Aneurysmal Dilatation: A Case Report. Acta Marisiensis Ser. Med..

[22] Najjar S.F. (2006). VASCULAR SURGERY-2 VOLUME SET, 6TH EDITION. Shock.

[23] Russu E., Mureșan A.V., Cordoș B.A., Copotoiu C., Cotoi O.S. (2016). Morpho-Pathological Review on the Healing of Synthetic Vascular Grafts. Acta Marisiensis Ser. Med..

[24] Eliza R., Vasile M.A., Andrei C.B., Simion C.O., Constantin C. (2015). Tissue Integration of Synthetic Grafts and the Impact of Soft-Tissue Infection—An Experimental Model. Acta Med. Marisiensis.

[25] Branchereau A., Rudondy P., Gournier J.-P., Espinoza H. (1990). The Albumin-Coated Knitted Dacron Aortic Prosthesis: A Clinical Study. Ann. Vasc. Surg..

[26] den Hoed P.T., Veen H.F. (1992). The Late Complications of Aorto-Ilio-Femoral Dacron Prostheses: Dilatation and Anastomotic Aneurysm Formation. Eur. J. Vasc. Surg..

[27] Greisler H.P. (1995). Characteristics and healing of vascular grafts. Vasc. Surg. Theory Pract..

[28] Halmaciu I., Arbănași E.M., Kaller R., Mureșan A.V., Arbănași E.M., Bacalbasa N., Suciu B.A., Cojocaru I.I., Runcan A.I., Grosu F. (2022). Chest CT Severity Score and Systemic Inflammatory Biomarkers as Predictors of the Need for Invasive Mechanical Ventilation and of COVID-19 Patients’ Mortality. Diagnostics.

[29] Arbănași E.M., Halmaciu I., Kaller R., Mureșan A.V., Arbănași E.M., Suciu B.A., Coșarcă C.M., Cojocaru I.I., Melinte R.M., Russu E. (2022). Systemic Inflammatory Biomarkers and Chest CT Findings as Predictors of Acute Limb Ischemia Risk, Intensive Care Unit Admission, and Mortality in COVID-19 Patients. Diagnostics.

[30] Kaller R., Arbănași E.M., Mureșan A.V., Voidăzan S., Arbănași E.M., Horváth E., Suciu B.A., Hosu I., Halmaciu I., Brinzaniuc K. (2022). The Predictive Value of Systemic Inflammatory Markers, the Prognostic Nutritional Index, and Measured Vessels’ Diameters in Arteriovenous Fistula Maturation Failure. Life.

[31] Mureșan A.V., Hălmaciu I., Arbănași E.M., Kaller R., Arbănași E.M., Budișcă O.A., Melinte R.M., Vunvulea V., Filep R.C., Mărginean L. (2022). Prognostic Nutritional Index, Controlling Nutritional Status (CONUT) Score, and Inflammatory Biomarkers as Predictors of Deep Vein Thrombosis, Acute Pulmonary Embolism, and Mortality in COVID-19 Patients. Diagnostics.

[32] Arbănași E.M., Mureșan A.V., Coșarcă C.M., Kaller R., Bud T.I., Hosu I., Voidăzan S.T., Arbănași E.M., Russu E. (2022). Neutrophil-to-Lymphocyte Ratio and Platelet-to-Lymphocyte Ratio Impact on Predicting Outcomes in Patients with Acute Limb Ischemia. Life.

[33] Melinte R.M., Arbănași E.M., Blesneac A., Zolog D.N., Kaller R., Mureșan A.V., Arbănași E.M., Melinte I.M., Niculescu R., Russu E. (2022). Inflammatory Biomarkers as Prognostic Factors of Acute Deep Vein Thrombosis Following the Total Knee Arthroplasty. Med. (Mex.).

[34] Russu E., Mureșan A.V., Arbănași E.M., Kaller R., Hosu I., Voidăzan S., Arbănași E.M., Coșarcă C.M. (2022). The Predictive Role of NLR and PLR in Outcome and Patency of Lower Limb Revascularization in Patients with Femoropopliteal Disease. J. Clin. Med..

[35] Arbănași E.M., Mureșan A.V., Arbănași E.M., Kaller R., Cojocaru I.I., Coșarcă C.M., Russu E. (2022). The Neutrophil-to-Lymphocyte Ratio’s Predictive Utility in Acute Pulmonary Embolism: Systematic Review. J. Cardiovasc. Emergencies.

[36] Niculescu R., Russu E., Arbănași E.M., Kaller R., Arbănași E.M., Melinte R.M., Coșarcă C.M., Cocuz I.G., Sabău A.H., Tinca A.C. (2022). Carotid Plaque Features and Inflammatory Biomarkers as Predictors of Restenosis and Mortality Following Carotid Endarterectomy. Int. J. Environ. Res. Public. Health.

[37] Mureșan A.V., Russu E., Arbănași E.M., Kaller R., Hosu I., Arbănași E.M., Voidăzan S.T. (2022). The Predictive Value of NLR, MLR, and PLR in the Outcome of End-Stage Kidney Disease Patients. Biomedicines.

[38] Mills J.L., Conte M.S., Armstrong D.G., Pomposelli F.B., Schanzer A., Sidawy A.N., Andros G. (2014). The Society for Vascular Surgery Lower Extremity Threatened Limb Classification System: Risk Stratification Based on Wound, Ischemia, and Foot Infection (WIfI). J. Vasc. Surg..

[39] King M.P., Blais R., Guidoin E., Prowse M., Marois C., Gosselin H.P., Noel. (1981). Polyethylene terephthalate (Dacron) vascular prostheses-material and fabric construction aspects. Biocompat. Clin. Implant. Mater..

[40] Davids L., Dower T., Zilla P. (1999). The lack of healing in conventional vascular grafts. Tissue Eng. Prosthet. Vasc. Grafts.

[41] Nunn D.B. (1999). Structural Failure of Dacron Arterial Grafts. Semin Vasc. Surg..

[42] Sottiurai V.S., Yao J.S.T., Flinn W.R., Batson R.C. (1983). Intimal Hyperplasia and Neointima: An Ultrastructural Analysis of Thrombosed Grafts in Humans. Surgery.

[43] Clowes A.W., Gown A.M., Hanson S.R., Reidy M.A. (1985). Mechanisms of Arterial Graft Failure. 1. Role of Cellular Proliferation in Early Healing of PTFE Prostheses. Am. J. Pathol.

[44] Scott S.M., Gaddy L.R., Sahmel R., Hoffman H. (1987). A Collagen Coated Vascular Prosthesis. J. Cardiovasc Surg. (Torino).

[45] Cziperle D.J., Joyce K.A., Tattersall C.W., Henderson S.C., Cabusao E.B., Garfield J.D., Kim D.U., Duhamel R.C., Greisler H.P. (1992). Albumin Impregnated Vascular Grafts: Albumin Resorption and Tissue Reactions. J. Cardiovasc Surg. (Torino).

[46] Akers D.L., Du Y.H., Kempczinski R.F. (1993). The Effect of Carbon Coating and Porosity on Early Patency of Expanded Polytetrafluoroethylene Grafts: An Experimental Study. J. Vasc. Surg..

[47] Prager M., Polterauer P., Böhmig H.-J., Wagner O., Fügl A., Kretschmer G., Plohner M., Nanobashvili J., Huk I. (2001). Collagen versus Gelatin-Coated Dacron versus Stretch Polytetrafluoroethylene in Abdominal Aortic Bifurcation Graft Surgery: Results of a Seven-Year Prospective, Randomized Multicenter Trial. Surgery.

[48] Arbănași E.-M., Russu E., Mureșan A.V., Arbănași E.-M. (2021). Late Rupture of a Thrombosed Aortic Abdominal Aneurysm—A Case Report. J. Cardiovasc. Emergencies.

[49] Robinson B.I., Fletcher J.P., Tomlinson P., Allen R.D.M., Hazelton S.J., Richardson A.J., Stuchbery K. (1999). A Prospective Randomized Multicentre Comparison of Expanded Polytetrafluoroethylene and Gelatin-Sealed Knitted Dacron Grafts for Femoropopliteal Bypass. Cardiovasc. Surg..

[50] Devine C., McCollum C. (2004). Heparin-Bonded Dacron or Polytetrafluorethylene for Femoropopliteal Bypass: Five-Year Results of a Prospective Randomized Multicenter Clinical Trial. J. Vasc. Surg..

[51] Post S., Kraus T., Müller-Reinartz U., Weiss C., Kortmann H., Quentmeier A., Winkler M., Husfeldt K.J., Allenberg J.R. (2001). Dacron vs Polytetrafluoroethylene Grafts for Femoropopliteal Bypass:A Prospective Randomised Multicentre Trial. Eur. J. Vasc. Endovasc. Surg..

[52] Green R.M., Abbott W.M., Matsumoto T., Wheeler J.R., Miller N., Veith F.J., Money S., Garrett H.E. (2000). Prosthetic Above-Knee Femoropopliteal Bypass Grafting: Five-Year Results of a Randomized Trial. J. Vasc. Surg..

[53] Robinson B.I., Fletcher J.P. (2003). The Australian and New Zealand Femoropopliteal Graft Trial Participants Fluoropolymer Coated Dacron or Polytetrafluoroethylene for Femoropopliteal Bypass Grafting: A Multicentre Trial. ANZ J. Surg..

[54] Jensen L.P., Lepäntalo M., Fossdal J.E., Røder O.C., Jensen B.S., Madsen M.S., Grenager O., Fasting H., Myhre H.O., Bækgaard N. (2007). Dacron or PTFE for Above-Knee Femoropopliteal Bypass. A Multicenter Randomised Study. Eur. J. Vasc. Endovasc. Surg..

